# Mortality, Severity, and Hospital Admission among COVID-19 Patients with ACEI/ARB Use: A Meta-Analysis Stratifying Countries Based on Response to the First Wave of the Pandemic

**DOI:** 10.3390/healthcare9020127

**Published:** 2021-01-28

**Authors:** Ahmad A. Alamer, Abdulaziz S. Almulhim, Ahmed A. Alrashed, Ivo Abraham

**Affiliations:** 1 Center for Health Outcomes and PharmacoEconomic Research, University of Arizona, 1295 N Martin Ave, Tucson, AZ 85721, USA; abraham@pharmacy.arizona.edu; 2 Department of Clinical Pharmacy, College of Pharmacy, Prince Sattam Bin Abdulaziz University, Alkharj 11942, Saudi Arabia; 3 Department of Pharmacy Practice, College of Clinical Pharmacy, King Faisal University, Al-Ahasa 31982, Saudi Arabia; asaalmulhim@kfu.edu.sa; 4 Pharmaceutical Service Department, Main Hospital, King Fahad Medical City, Riyadh 11564, Saudi Arabia; dr.azizsaleh.aa@gmail.com; 5 Department of Pharmacy Practice and Science, College of Pharmacy, University of Arizona, Tucson, AZ 85721, USA

**Keywords:** angiotensin-converting enzyme inhibitor, angiotensin II receptor blocker, Covid-19, mortality, disease severity, hospital admission

## Abstract

Background: The use of angiotensin-converting enzyme inhibitors (ACEIs) or angiotensin receptor blockers (ARBs) is controversial for treating COVID-19 patients. We aimed to estimate pooled risks of mortality, disease severity, and hospitalization associated with ACEI/ARB use and stratify them by country and country clusters. Methods: We conducted a search in various databases through 4 July 2020 and then applied random-effects models to estimate pooled risks (OR_p_) across stratifications by country cluster. Clusters were chosen to reflect outbreak times (China followed by Korea/Italy, others subsequently) and mobility restrictions (China and Denmark/France/Spain with stricter lockdowns than the UK/US). Results: Overall analysis showed no increase in mortality; however, a statistical increase in mortality was seen in the US/UK cluster with OR_p_ = 1.28 [95% CI = 1.04; 1.56] and a decrease in China with OR_p_ = 0.65 [95% CI = 0.43; 0.96] and France with OR = 0.31 [95% CI = 0.14; 0.69]. Severity and hospitalization were not statistically significant in the analysis; however, several associations were seen in specific countries but not in country clusters. Conclusion: The country-cluster meta-analysis provided a reasonable explanation for COVID-19 mortality among ACEI/ARB users. The analysis did not explain differences in severity and suggested the involvement of other factors. Hospitalization findings among ACEI/ARB users may be considered informative as they may have been subjected to clinical decisions and hospital-bed availability.

## 1. Background

Scientists have attempted to model the impact of non-pharmaceutical interventions (NPIs) on the global pandemic caused by the coronavirus beginning in 2019. NPIs may include contact tracing, increased testing, social distancing, wearing masks, and extreme measures such as complete lockdowns and banning public gatherings [1]. The most extreme example of an NPI was the national lockdown in Europe that included Italy, Spain, France, and Denmark, among other EU nations, and in China, which aimed to change the trajectory of the pandemic. This strategy was shown to be effective in reducing the time-varying reproduction number (Rt) of COVID-19—”an epidemiological quantity that represents the average number of infections generated at time (*t*) by each infected case over the course of their infection”—almost immediately after implementation [1]. By contrast, given the adverse economic impact of such strategies, few countries adopted the “herd immunity” strategy during the pandemic or imposed a delayed implementation of the lockdown [2,3]. The literature suggests that low levels of national preparedness and governmental responses can increase the likelihood of an overwhelmed healthcare system that could result in adverse health outcomes for the most vulnerable patients in a society [4].

With the recent concern regarding angiotensin-converting enzyme inhibitors (ACEI) and angiotensin II receptor blockers (ARB), there has been speculation that their use could increase the risk of exacerbating COVID-19 infections by upregulating ACE2 expression [5,6]. However, it has also been hypothesized that these could yield favorable outcomes [7]. These conflicting opinions are supported by published human studies, as we will discuss in this study.

Interestingly, many diseases such as hypertension, diabetes, renal, and cardiovascular diseases share a common indication for ACEI/ARB [5]. Among other factors, the association of ACEIs/ARBs with increased risk of COVID-19 requires further evaluation in this subset of vulnerable patients [5]. 

Beyond the theoretical risk in these patients, we attempted to evaluate whether or not specific countries and country clusters were successful in mitigating the risk of worse COVID-19 outcomes in ACEI/ARB users compared to non-ACEI/ARB users during their first wave of the pandemic. The results of this study would help to explain several of the discrepancies seen in COVID-19 outcomes associated with ACEIs/ARBs in the published literature. Therefore, the primary objective was to estimate the pooled risks of mortality and disease severity associated with ACEI/ARB use during the first wave of the pandemic. Exploratorily, we also estimated the pooled risk of hospitalization with due caution because of factors independent of COVID-19. 

## 2. Methods

### 2.1. Literature Search and Data Extraction

We complemented an amended [8] meta-analysis and a living systematic review [9], [10] with an updated search through 4 July 2020 in the PubMed, Cochrane, and medRxiv.org databases. A PRISMA flowchart is shown in Figure S1 in the Supplementary Materials. The following modified population, intervention, comparison, outcome, study type (PICOS) criteria [11]. (1) Population: patients of any age who tested positive for COVID-19; (2) intervention: ACEs or ARBs; (3) comparison: placebo or active control; (4) outcomes: mortality, disease-severity, and hospital admission; (5) study type: controlled and non-controlled. The definition for COVID-19 severity may include the following: National Health Commission of the People’s Republic of China [12], WHO severity definition [13], requiring intensive care unit (ICU) care, Infectious Disease Society of America (IDSA) pneumonia severity [14], requiring mechanical ventilation or the development of Acute Respiratory Distress Syndrome (ARDS). Studies published a language other than English were excluded.

Two authors (Ahmad A. Alamer; Abdulaziz S. Almulhim) screened publications and extracted odds ratios (OR). For studies not reporting ORs but including adequate data, we estimated the crude OR (unadjusted). Each unadjusted OR was calculated with its standard error and 95% confidence interval according to Altman et al. [15]. In the case of zero events, we estimated the odds ratio with 0.5 correction in accordance with Deeks and Higgins’s recommendations [16]. In studies where adjusted odds ratios were reported, we used the reported estimates.

### 2.2. Data Synthesis and Analysis

Using R Core Team (2020) software (R Foundation for Statistical Computing, Version 4.0.1, Vienna, Austria) and the meta package [17], we applied random-effects models to estimate pooled risks (OR_p_) across all studies and then stratified the analysis by studies with and without statistical adjustment. The package uses the generic inverse variance method for the meta-analysis and requires the estimates (OR) and their standard errors as inputs to calculate the pooled estimates in accordance with Borenstein et al. [18].

Q-test and *I*^2^ were used to quantify heterogeneity of the included studies. An *I*^2^ > 50% indicates significant heterogeneity. We conducted Egger’s test and produced funnel plots to assess publication bias using the same package. A *p* value < 0.05 considered to be statistically significant. We performed sensitivity subgroup analyses by country and country clusters. The clusters were chosen to reflect time of outbreaks (China first, Korea/Italy next, others subsequently) and mobility restrictions (China and Denmark/France/Spain with stricter lockdowns than the UK/US). Quality assessment was carried out using the Newcastle–Ottawa Scale (NOS) [19].

## 3. Results

**Figure 1:**
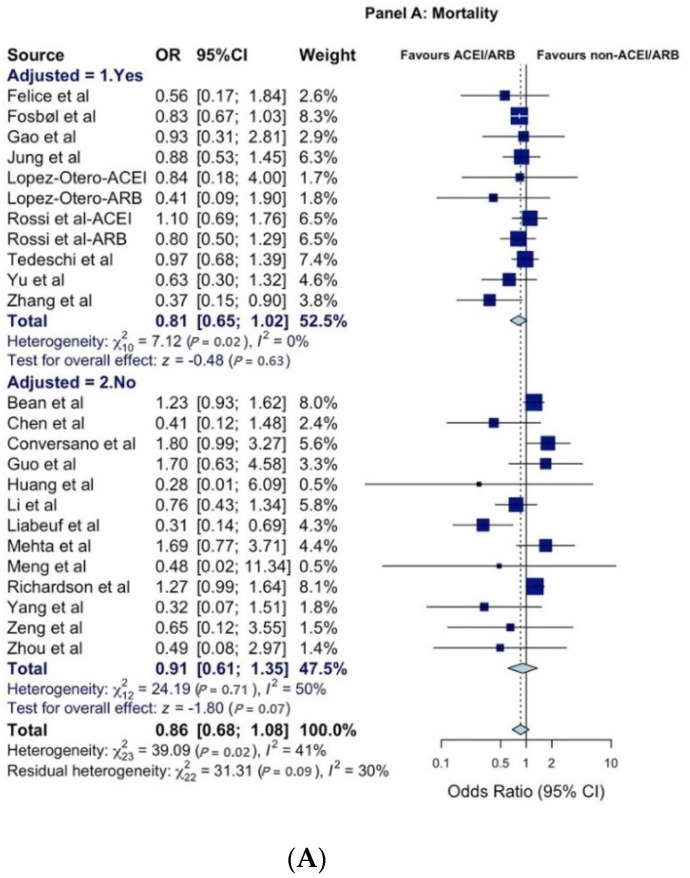

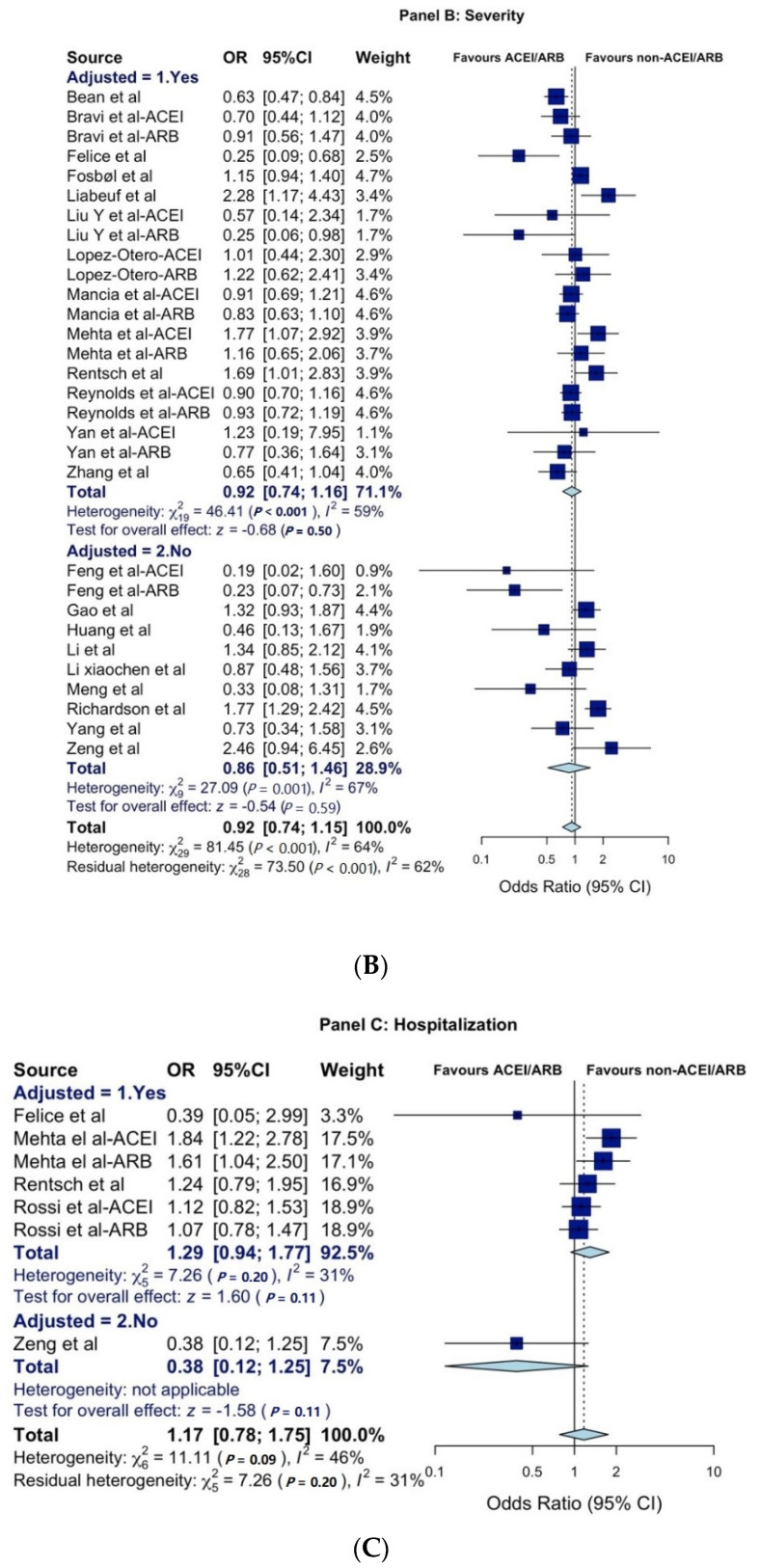
Forest plots for (**A**) mortality, (**B**) severity of COVID-19 disease, and (**C**) hospitalization. All studies were published in 2020. All citations included in the Supplementary Material Appendix to Table S1. The size of squares is proportional to the weight of each study. Horizontal lines indicate the 95% CI of each study; diamonds indicate the pooled estimate with 95% CI. Abbreviations: CI: confidence interval; OR: odds ratio. ACEI: Angiotensin-converting enzyme inhibitors. ARB: Angiotensin II receptor blockers.

**Figure 2:**
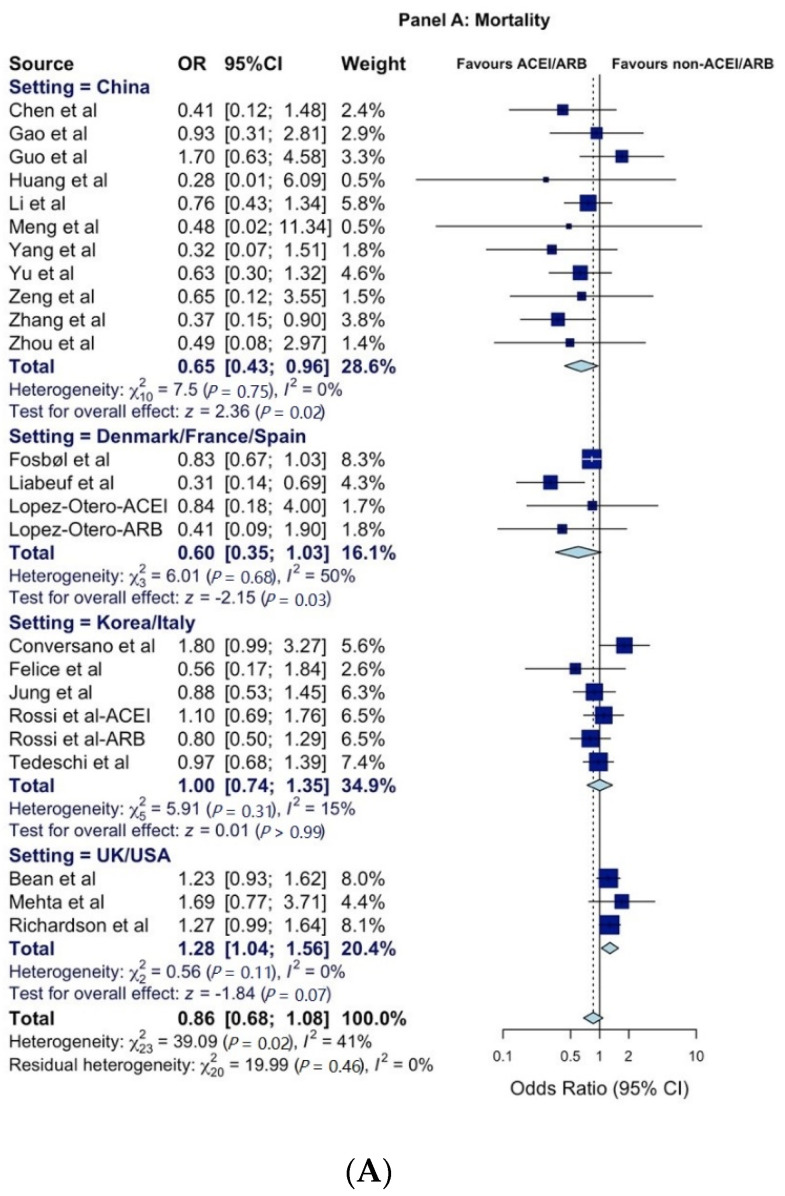

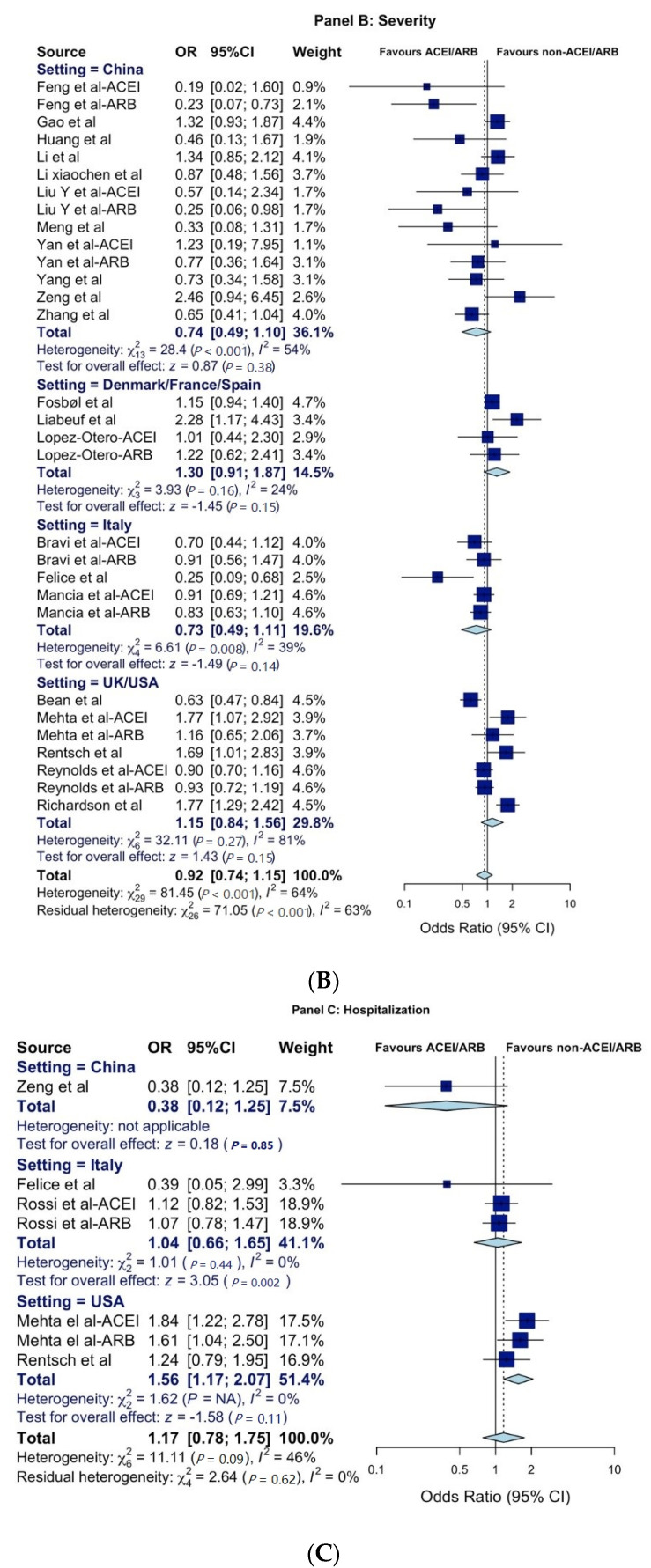
Forest plots for country clusters (**A**) mortality, (**B**) severity of COVID-19 disease, and (**C**) hospitalization. All studies were published in 2020. All citations included in the Supplementary Materials file Appendix to Table S1. The size of squares is proportional to the weight of each study. Horizontal lines indicate the 95% CI of each study; diamonds, the pooled estimate with 95% CI. Abbreviations: CI: confidence interval; OR: odds ratio. ACEI: Angiotensin-converting enzyme inhibitors. ARB: Angiotensin II receptor blockers.

A total of 30 publications reporting 61 estimates (*k*) for mortality, disease-severity, and/or hospitalization analyses were included (Table S1 and Figure S1 in the Supplementary Materials). The OR_p_ for mortality (*k* = 24) was statistically non-significant at 0.86 (95% CI = 0.68–1.08) and remaining non-significant when stratified by studies that reported adjusted or non-adjusted estimates (Figure 1A). The subgroup sensitivity analyses by country and country clusters for the mortality outcome was not significant, except for China and France, where a decrease was seen, and the UK/US cluster, where an increase in mortality risk in association with ACEI/ARB exposure was observed (Table 1; Figure 2A). The OR_p_ for COVID-19 disease severity (*k* = 30) was 0.92 (95% CI = 0.74–1.15) and remained statistically non-significant when stratified by studies with or without adjustments (Figure 1B).

The single French study reported a statistically significant increase in COVID-19 severity with ACEI/ARB use, while the one British study related a significant decrease in severe COVID-19 disease risk in association with ACEI/ARB use (Figure 2B and Table 1). The OR_p_ for hospitalization (*k* = 7) was 1.17 (95% CI = 0.78–1.75) and remained statistically non-significant in studies with or without adjustment (Figure 1C and Table 1). The association of ACEI/ARB use, and hospitalization risk was non-significant for China and Italy but significant for the US (Figure 2C and Table 1). Heterogeneity was low (*I*^2^ < 30% = 11) to moderate (*I^2^* 30%–60% = 8) with some high (*I*^2^ > 60% = 4). Funnel plots and Egger’s tests were significant for mortality (*p* = 0.04) but not for disease severity (*p* = 0.216) and hospitalization (*p* = 0.337), as shown in Figure S2.

## 4. Discussion

With a verifiable total of 43,829 patients in the analysis, including 11,166 exposed to ACEI/ARB, our meta-analysis consistently revealed the absence of an association between ACEI/ARB use and mortality, disease severity, and hospitalization risk in COVID-19, a finding to be validated as further evidence accumulates. The country cluster sensitivity analysis explained several of the differences seen in the mortality outcome. The UK/US cluster revealed an increased risk of mortality. The included studies for this cluster reported non-adjusted OR for the mortality outcome (see Table S1). The study by Richardson et al. was a large case series in New York conducted between March 1 and April 4, 2020, dates that coincide with that state’s first wave of the pandemic prior to extreme measures such as stay-at-home orders being taken [20,21]. A similar pattern emerges with the UK study by Bean et al. in which the country experienced their first wave prior to lockdown measures [1,21,22,23]. Studies in France, Italy, China, Denmark, Spain and Korea were also conducted in their first wave; however, in contrast, during that time they had already implemented multiple NPI strategies (including a national lockdown) to mitigate the impact of the pandemic [1,24,25]. Based on our analysis, two of these countries (France and China) saw a decrease in mortality among ACEI/ARB users (shown in Figure 2A). It would be difficult to determine whether this effect was due to an underlying mechanism of ACEI/ARB protection or if it was the result of very strict NPIs having been executed. Many of the included studies controlled for potential confounders that can affect the outcomes (see Table S1). With cautious interpretation, among many other factors, this may suggest that countries with much stricter NPI policies may have been successful in mitigating the risk of overall mortality among ACEI/ARB users. This finding should be viewed only as a signal as the majority of the studies included in the pooled synthesis were observational in nature.

The subgroup sensitivity analyses by country clusters did not explain the heterogeneity in the disease-severity endpoint very well which was evident by *I^2^* > 50% in subgroup analysis by countries such as China and US/UK cluster. The included studies defined COVID-19 severity differently and that may explain the seen heterogeneity. Many of the included studies defined COVID-19 severity according to the National Health Commission of China which is similar to the WHO definition [12,13]. The second frequent definition was ICU care as in indication for COVID-19 severity. Only two studies used IDSA pneumonia severity definition (see Table S1 for definitions). Indeed, harmonization of disease-severity definitions may strengthen future studies and meta-analyses; however, more importantly, some researchers suggest the existence of ACE-2 overexpression polymorphism as a potential explanation for the severity of the COVID-19 presentation [26]. The country-cluster analysis is not well-suited to answer this question, and more studies among different ethnic groups are needed. For example, in a large cohort in the UK, it was found that Blacks being treated with ACEIs/ARBs were more susceptible to COVID-19 compared to Whites [27]. Whether this ethnic difference is real is yet to be determined, and the discordant country-specific results are points for future attention as more data start to accumulate. Lastly, the sensitivity analysis by country revealed more hospitalizations in the US among ACEI/ARB users. We believe that the hospitalization findings should be considered informative, at best, and not very reliable as these are influenced by clinical decisions and hospital-bed availability and not based on objective criteria.

An advantage of this current analysis is that we captured published studies from early stages of the pandemic in each country. Therefore, we think that we were able to estimate the risk of mortality during the implementation of NPI measures (especially national lockdowns) in a number of countries. The South Korean national response was a highly successful model for handling the pandemic. With aggressive measures that included contact tracing to prevent community transmissibility, South Korea reported the largest numbers of cases in the first two months of the pandemic. In this current analysis there was no signal of increased mortality among ACEI/ARB users in South Korea [25]. It should be emphasized this finding is based on one observational study from the country that was adjusted for potential confounders.

History of previous pandemics such The Spanish flu in 1918 taught us that subsequent waves (second and third) is likely to occur. The second wave of the Spanish flu was believed to be caused by a mutated virus [28]. There are several proposed mechanisms for a third wave such as increase viral transmissibility due to seasonal changes, changes in social mixing (such as holidays, schools’ closure and re-opening), viral mutation and emergence of escape variant mutation [29]. These pandemic waves and the potential of catastrophic consequences for high risk patients can be mitigated by initiating vaccination programs [30]. This would be possible with the recent introduction of highly effective vaccines in a wrap speed mission to control the pandemic [31,32].

## 5. Conclusions

In summary, our meta-analysis of studies accrued to 4 July 2020 suggests no evidence of an association of ACEI/ARB exposure with mortality, COVID-19 disease severity, or hospitalization. The country-cluster meta-analysis provided a reasonable explanation for differences in the mortality outcome, while it failed to explain the severity outcome. More studies of ACEIs/ARBs and COVID-19 severity outcomes in different populations are needed. The association of ACEIs/ARBs with mortality outcomes may be related in part to waves of infection; non-pharmaceutical measures such as mobility restrictions, contact tracing, and increased testing, and progress in vaccines and therapeutic treatments.

## Figures and Tables

**Table 1:** Overall and stratified pooled risk estimates for mortality, severity, and hospitalization ^1^.

Outcome/Source	Studies ^2^	OR_p_ [95% CI] ^3^	*I^2^* (if *k* ≥ 2)
**Mortality**			
All reports ^4^	24	0.86 [0.68; 1.08]	41%
Adjusted	11	0.81 [0.65; 1.02]	0%
Unadjusted ^5^	13	0.91 [0.61; 1.35]	50%
By country/cluster			
China	11	0.65 [0.43; 0.96]	0%
Korea	1	0.88 [0.53; 1.45]	-
Italy	5	1.03 [0.71; 1.46]	29%
Denmark	1	0.83 [0.67; 1.03]	-
France	1	0.31 [0.14; 0.69]	-
Spain	2	0.58 [0.19; 1.81]	0%
UK	1	1.23 [0.93; 1.62]	-
US	2	1.32 [0.99; 1.75]	0%
Korea/Italy	6	1.00 [0.74; 1.36]	15%
Denmark/France/Spain	4	0.60 [0.35; 1.03]	50%
UK/US	3	1.28 [1.04; 1.56]	0%
**Severity**			
All reports ^4^	30	0.92 [0.74; 1.15]	64%
Adjusted	20	0.92 [0.74; 1.16]	59%
Unadjusted ^5^	10	0.90 [0.61; 1.33]	67%
By country/cluster			
China	14	0.74 [0.50; 1.10]	54%
Italy	5	0.74 [0.49; 1.11]	40%
Denmark	1	1.15 [0.94; 1.40]	-
France	1	2.28 [1.17; 4.43]	-
Spain	2	1.13 [0.67; 1.91]	0%
UK	1	0.63 [0.47; 0.84]	-
US	6	1.27 [0.96; 1.66]	73%
Denmark/France/Spain	4	1.30 [0.91; 1.87]	24%
UK/US	7	1.15 [0.84; 1.56]	81%
**Hospitalization**			
All reports ^4^	7	1.17 [0.78; 1.75]	46%
Adjusted	6	1.29 [0.94; 1.77]	31%
Unadjusted ^5^	1	0.38 [0.12; 2.91]	-
By country			
*China*	1	0.38 [0.12; 1.25]	-
*Italy*	3	1.04 [0.66; 1.65]	0%
*US*	3	1.56 [1.17; 2.07]	0%

^1^ Patients (ACEI/ARB vs. non-ACEI/ARB) in analyses: mortality: 4145 vs. 14,996; severity: 8168 vs. 28,976; hospitalization: 1374 vs. 8138 (numbers are approximate because of inconsistent reporting as some studies did not report the exact number of patients on ACEI/ or ARB for specific outcomes); ^2^ Number of studies included in the meta-analysis. Studies with separate estimates for both ACEI and ARB are counted separately; ^3^ Random effect models were used for all analyses. OR_p_ was estimated if ≥2 studies and OR if only 1 study in the analysis; ^4^ Combined analysis of adjusted and unadjusted odds ratios; ^5^ Crude OR calculated for studies reporting adequate data; ACEI: angiotensin-converting enzyme inhibitors; ARB: angiotensin II receptor blockers; CI: confidence interval; *k*: number of estimates in analysis; OR: odds ratio; OR_p_: pooled odds ratio.

## Data Availability

The datasets supporting the conclusions of this article are included in the Supplementary Materials.

## Supplementary Materials

The following are available online at https://www.mdpi.com/2227-9032/9/2/127/s1, Figure S1: PRISMA flowchart. Figure S2: Funnel plots (A) mortality, (B) severity of COVID-19 disease, and (C) hospitalization, Table S1: Corpus of studies included in meta-analysis (search closing date of 4 July 2020; citations in Appendix to this Table S1).

## Abbreviations

NPIs	Non-pharmaceutical interventions
COVID-19	Novel coronavirus
Rt	Reproduction number
t	Time
*k*	estimate
ACEIs	Angiotensin-converting enzyme inhibitors
ARBs	Angiotensin-receptor blockers
ACE2	Angiotensin-converting enzyme-2
PRISMA	Preferred Reporting Items for Systematic Reviews and Meta-Analyses
OR	Odds ratio
ORp	Pooled odds ratio
CI	Confidence Interval
NOS	Newcastle–Ottawa Scale
ICU	Intensive care unit
