# Supplementary material for: Mortality, Severity, and Hospital Admission among COVID-19 Patients with ACEI/ARB Use: A Meta-Analysis Stratifying Countries Based on Response to the First Wave of the Pandemic
Source: Healthcare (Basel). 2021 Jan 28;9(2):127. doi: 10.3390/healthcare9020127 (PMC7912160; doi:10.3390/healthcare9020127)

healthcare

Supplementary Online Content

Table S1. Corpus of studies included in meta-analysis (search closing date of 4 July 2020.

Appendix to Table S1: Publications included in the meta-analysis

Fig. S1: PRISMA flowchart.

Fig. S2: Funnel plots (A) mortality, (B) severity of COVID-19 disease, and (C) hospitalization.

Table S1. Corpus of studies included in meta-analysis (search closing date of 4 July 2020; citations in Appendix to this Table).

Study	Country	Design	Study Period	ACEI/ARB	Non-ACEI/ARB	Outcomes of interest	Statistical adjustments	NOS
Bean et al[1]	UK	Multi-center retrospective study	1st Mar to 13th Apr, 2020	399	801	Mortality<br>Severity (death or organ support within 21-days of symptom onset)	Mortality: none<br>Severity: age, sex, comorbidities	7
Bravi et al[2]	Italy	Population-based case-control retrospective study	Up to 24th Apr, 2020	450	1152	Severity (requiring ICU or causing death)	Severity: age, gender, diabetes, major cardiovascular diseases, COPD, cancer, renal diseases	7
Chen et al[3]	China	Single-center retrospective study	1st Jan to Mar 17th, 2020	32	39	Mortality	None	5
Conversano et al[4]	Italy	Single-center retrospective study	27th Feb to 17th Mar, 2020	-	-	Mortality	None	5
Felice et al[5]	Italy	Single-center retrospective study	9th Mar to 31st Mar 2020	82	51	Mortality<br>Severity (requiring semi ICU or ICU)<br>Hospitalization	Mortality, severity, hospitalization: age, gender, BMI, days with symptoms, previous cardiovascular	8
Feng et al[6]	China	Multi-center retrospective study	1st Jan to 15th Feb 2020	35	62	Severity (National Health Commission of China definition)	None	5

Fosbøl et al[7]	Denmark	National registry retrospective cohort study	1st Feb to 4th May, 2020	895	3585	Mortality Severity (unclear definition)	Mortality, severity: age, sex, comorbidities	9
Gao et al[8]	China	Single-center retrospective observational study	5th Feb to 15th Mar, 2020	183	527	Mortality Severity (unclear definition)	Mortality, severity: propensity score matched for age, sex, diabetes, insulin-treated diabetes, myocardial infarction, underwent PCI/CABG, renal failure, stroke, heart failure, COPD.	8
Guo et al[9]	China	Single-center retrospective study	23th Jan to 23th Feb, 2020	19	168	Mortality	None	4
Huang et al[10]	China	Single-center retrospective study	7th Feb to 3rd Mar, 2020	20	30	Mortality Severity (National Health Commission of China definition)	None	4
Jung et al[11]	Korea	Retrospective observational data from nationwide database	Up to 8th Apr, 2020.	377	1577	Mortality (in-hospital death)	Mortality: age, sex, Charlson Comorbidity Index, immunosuppression	7
Li et al[12]	China	Single-center retrospective case series	15th Jan to 15th Mar, 2020	115	247	Mortality Severity (National Health Commission of China definition)	None	4
Liabeuf et al[13]	France	Single-center retrospective study	Feb 28th to 30th Mar, 2020	96	172	Mortality Severity (admission to ICU)	Mortality: none Severity: comorbidities	4

Li Xiaochen et al[14]	China	Single-center ambispective cohort study	Jan 26th to Feb 5th, 2020	42	503	Severity (IDSA Pneumonia severity)	None	4
Liu Y et al[15]	China	Multi-center retrospective study	Jan 11th to Feb 5th, 2020	12	34	Severity (National Health Commission of China definition)	Severity: sex	6
Lopez-Otero[16]	Spain	Single-center retrospective study	Mar 10th to April 6th, 2020	210	755	Mortality Severity (ICU admissions)	Mortality, severity: propensity score matching	8
Mancia et al[17]	Italy	Population-based case–control study	Feb 21th to Mar 11th, 2020	2896	12506	Severity (defined as critical or fatal disease)	Severity: propensity score matching	9
Mehta et al[18]	USA	Multi-center retrospective study	Mar 8th to Apr 12th, 2020	211	1494	Mortality Severity (admission to ICU) Hospitalization	Mortality: none Severity, hospitalization: propensity score matching	8
Meng et al[19]	China	Single-center retrospective study	Jan 11th to Feb 23rd, 2020	17	25	Mortality Severity (National Health Commission of China definition)	None	4
Richardson et al[20]	USA	Population-based retrospective case series study	Mar 1st to Apr 4th, 2020	413	953	Mortality Severity (ICU care)	None	4
Rentsch et al[21]	USA	Population-based retrospective cohort	Feb 8th and Mar 30th, 2020	1532	2247	Severity (ICU admission) Hospitalization	Severity, hospitalization: age, race, comorbid conditions, and Veterans Aging Cohort Study Index of physiologic injury	9

Reynolds et al[22]	USA	Multi-center retrospective cohort study	Mar 1st to Apr 15th, 2020	1774	439	Severity (defined as intensive care, mechanical ventilation, or death)	Propensity score matching	9
Rossi et al[23]	Italy	Population-based prospective cohort study	Feb 27th Up to Apr 8th	818	4488	Mortality Hospitalization	Mortality, severity: age, sex, Charlson Comorbidity Index	8
Tedeschi et al[24]	Italy	Multi-center prospective cohort study	Feb 1st to Apr 4th, 2020	175	136	Mortality	Mortality: age, gender, presence of comorbidities	8
Yang et al[25]	China	Single-center retrospective study	Jan 5th to Feb 22nd, 2020	43	83	Mortality Severity (National Health Commission of China definition)	None	4
Yan et al[26]	China	Population-based retrospective case-control study	Jan 10th to Feb 28th, 2020	-	-	Severity (National Health Commission of China definition)	Severity: age, sex, BMI	4
Yu et al[27*]	China	Multi-center retrospective cohort study	Jan 17th to Feb 19th, 2020	103	173	Mortality	Mortality: sex, age, smoking, symptoms, chronic diseases	9
Zeng et al[28]	China	Single-center retrospective study	Jan 5th to Mar 8th, 2020	28	47	Mortality Severity (IDSA Pneumonia severity) Hospitalization	None	4
Zhang et al[29]	China	Multi-center retrospective study	Dec 31st to Feb 20th, 2020	174	348	Mortality Severity (ARDS)	Mortality, severity: propensity score matching	9
Zhou et al[30]	China	Single-center retrospective study	Jan 25th to Feb 20th, 2020	15	21	Mortality	None	4

* Data were not reported separately but merged into the Zhang et al (2020) meta-analysis. Data included here are as reported in the meta-analysis.

Abbreviations

ACEI: angiotensin converting enzyme inhibitors

ARB: Angiotensin II receptor blockers

ARDS: Acute respiratory distress syndrome

BMI: Body mass index

CABG: Coronary artery bypass surgery

COPD: Chronic obstructive pulmonary disease

ICU: Intensive care unit

IDSA: Infectious Diseases Society of America

PCI: Percutaneous coronary intervention

NOS: Newcastle-Ottawa Scale

Appendix to Table S1: Publications included in the meta-analysis

References:

1. Bean DM, Kraljevic Z, Searle T, et al. ACE-inhibitors and Angiotensin-2 Receptor Blockers are not associated with severe SARS-COVID19 infection in a multi-site UK acute Hospital Trust. *Eur J Heart Fail.* 2020.
2. Bravi F, Flacco ME, Carradori T, et al. Predictors of severe or lethal COVID-19, including Angiotensin Converting Enzyme inhibitors and Angiotensin II Receptor Blockers, in a sample of infected Italian citizens. *PLoS One.* 2020;15(6):e0235248.
3. Chen Y, Yang D, Cheng B, et al. Clinical Characteristics and Outcomes of Patients With Diabetes and COVID-19 in Association With Glucose-Lowering Medication. *Diabetes Care.* 2020;43(7):1399-1407.
4. Andrea C, Francesco M, Antonio N, et al. Renin-Angiotensin-Aldosterone System Inhibitors and Outcome in Patients With SARS-CoV-2 Pneumonia: A Case Series Study. *Hypertension.* 2020;76(2):e10-e12.
5. Felice C, Nardin C, Di Tanna GL, et al. Use of RAAS inhibitors and risk of clinical deterioration in COVID-19: results from an Italian cohort of 133 hypertensives. *Am J Hypertens.* 2020.
6. Feng Y, Ling Y, Bai T, et al. COVID-19 with Different Severities: A Multicenter Study of Clinical Features. *Am J Respir Crit Care Med.* 2020;201(11):1380-1388.
7. Fosbøl EL, Butt JH, Østergaard L, et al. Association of Angiotensin-Converting Enzyme Inhibitor or Angiotensin Receptor Blocker Use With COVID-19 Diagnosis and Mortality. *JAMA.* 2020.
8. Gao C, Cai Y, Zhang K, et al. Association of hypertension and antihypertensive treatment with COVID-19 mortality: a retrospective observational study. *Eur Heart J.* 2020;41(22):2058-2066.
9. Guo T, Fan Y, Chen M, et al. Cardiovascular Implications of Fatal Outcomes of Patients With Coronavirus Disease 2019 (COVID-19). *JAMA Cardiol.* 2020.
10. Huang Z, Cao J, Yao Y, et al. The effect of RAS blockers on the clinical characteristics of COVID-19 patients with hypertension. *Ann Transl Med.* 2020;8(7):430.
11. Jung SY, Choi JC, You SH, Kim WY. Association of renin-angiotensin-aldosterone system inhibitors with COVID-19-related outcomes in Korea: a nationwide population-based cohort study. *Clin Infect Dis.* 2020.
12. Li J, Wang X, Chen J, Zhang H, Deng A. Association of Renin-Angiotensin System Inhibitors With Severity or Risk of Death in Patients With Hypertension Hospitalized for Coronavirus Disease 2019 (COVID-19) Infection in Wuhan, China. *JAMA Cardiol.* 2020.
13. Liabeuf S, Moragny J, Bennis Y, et al. Association between renin-angiotensin system inhibitors and COVID-19 complications. *Eur Heart J Cardiovasc Pharmacother.* 2020.
14. Li X, Xu S, Yu M, et al. Risk factors for severity and mortality in adult COVID-19 inpatients in Wuhan. *J Allergy Clin Immunol.* 2020;146(1):110-118.
15. Liu Y, Huang F, Xu J, et al. Anti-hypertensive Angiotensin II receptor blockers associated to mitigation of disease severity in elderly COVID-19 patients. *medRxiv.* 2020:2003.2020.20039586.
16. López-Otero D, López-Pais J, Cacho-Antonio CE, et al. Impact of angiotensin-converting enzyme inhibitors and angiotensin receptor blockers on COVID-19 in a western population. CARDIOVID registry. *Rev Esp Cardiol (Engl Ed).* 2020.
17. Mancia G, Rea F, Ludergnani M, Apolone G, Corrao G. Renin–Angiotensin–Aldosterone System Blockers and the Risk of Covid-19. *New England Journal of Medicine.* 2020;382(25):2431-2440.

18. Mehta N, Kalra A, Nowacki AS, et al. Association of Use of Angiotensin-Converting Enzyme Inhibitors and Angiotensin II Receptor Blockers With Testing Positive for Coronavirus Disease 2019 (COVID-19). *JAMA Cardiol.* 2020.
19. Meng J, Xiao G, Zhang J, et al. Renin-angiotensin system inhibitors improve the clinical outcomes of COVID-19 patients with hypertension. *Emerg Microbes Infect.* 2020;9(1):757-760.
20. Richardson S, Hirsch JS, Narasimhan M, et al. Presenting Characteristics, Comorbidities, and Outcomes Among 5700 Patients Hospitalized With COVID-19 in the New York City Area. *JAMA.* 2020.
21. Rentsch CT, Kidwai-Khan F, Tate JP, et al. Covid-19 Testing, Hospital Admission, and Intensive Care Among 2,026,227 United States Veterans Aged 54-75 Years. *medRxiv.* 2020.
22. Reynolds HR, Adhikari S, Pulgarin C, et al. Renin-Angiotensin-Aldosterone System Inhibitors and Risk of Covid-19. *N Engl J Med.* 2020;382(25):2441-2448.
23. Giorgi Rossi P, Marino M, Formisano D, Venturelli F, Vicentini M, Grilli R. Characteristics and outcomes of a cohort of SARS-CoV-2 patients in the Province of Reggio Emilia, Italy. *medRxiv.* 2020:2020.2004.2013.20063545.
24. Tedeschi S, Giannella M, Bartoletti M, et al. Clinical Impact of Renin-angiotensin System Inhibitors on In-hospital Mortality of Patients With Hypertension Hospitalized for Coronavirus Disease 2019. *Clinical Infectious Diseases.* 2020.
25. Yang G, Tan Z, Zhou L, et al. Effects of Angiotensin II Receptor Blockers and ACE (Angiotensin-Converting Enzyme) Inhibitors on Virus Infection, Inflammatory Status, and Clinical Outcomes in Patients With COVID-19 and Hypertension: A Single-Center Retrospective Study. *Hypertension.* 2020;76(1):51-58.
26. Yan H, Valdes AM, Vijay A, et al. Role of Drugs Affecting the Renin-Angiotensin-Aldosterone System on Susceptibility and Severity of COVID-19: A Large Case-Control Study from Zheijang Province, China. *medRxiv.* 2020:2020.2004.2024.20077875.
27. Zhang X, Yu J, Pan LY, Jiang HY. ACEI/ARB use and risk of infection or severity or mortality of COVID-19: A systematic review and meta-analysis. *Pharmacol Res.* 2020;158:104927.
28. Zeng Z, Sha T, Zhang Y, et al. Hypertension in patients hospitalized with COVID-19 in Wuhan, China: A single-center retrospective observational study. *medRxiv.* 2020:2020.2004.2006.20054825.
29. Zhang P, Zhu L, Cai J, et al. Association of Inpatient Use of Angiotensin-Converting Enzyme Inhibitors and Angiotensin II Receptor Blockers With Mortality Among Patients With Hypertension Hospitalized With COVID-19. *Circ Res.* 2020;126(12):1671-1681.
30. Zhou X, Zhu J, Xu T. Clinical characteristics of coronavirus disease 2019 (COVID-19) patients with hypertension on renin-angiotensin system inhibitors. *Clin Exp Hypertens.* 2020;42(7):656-660.

Fig. S1: PRISMA flowchart

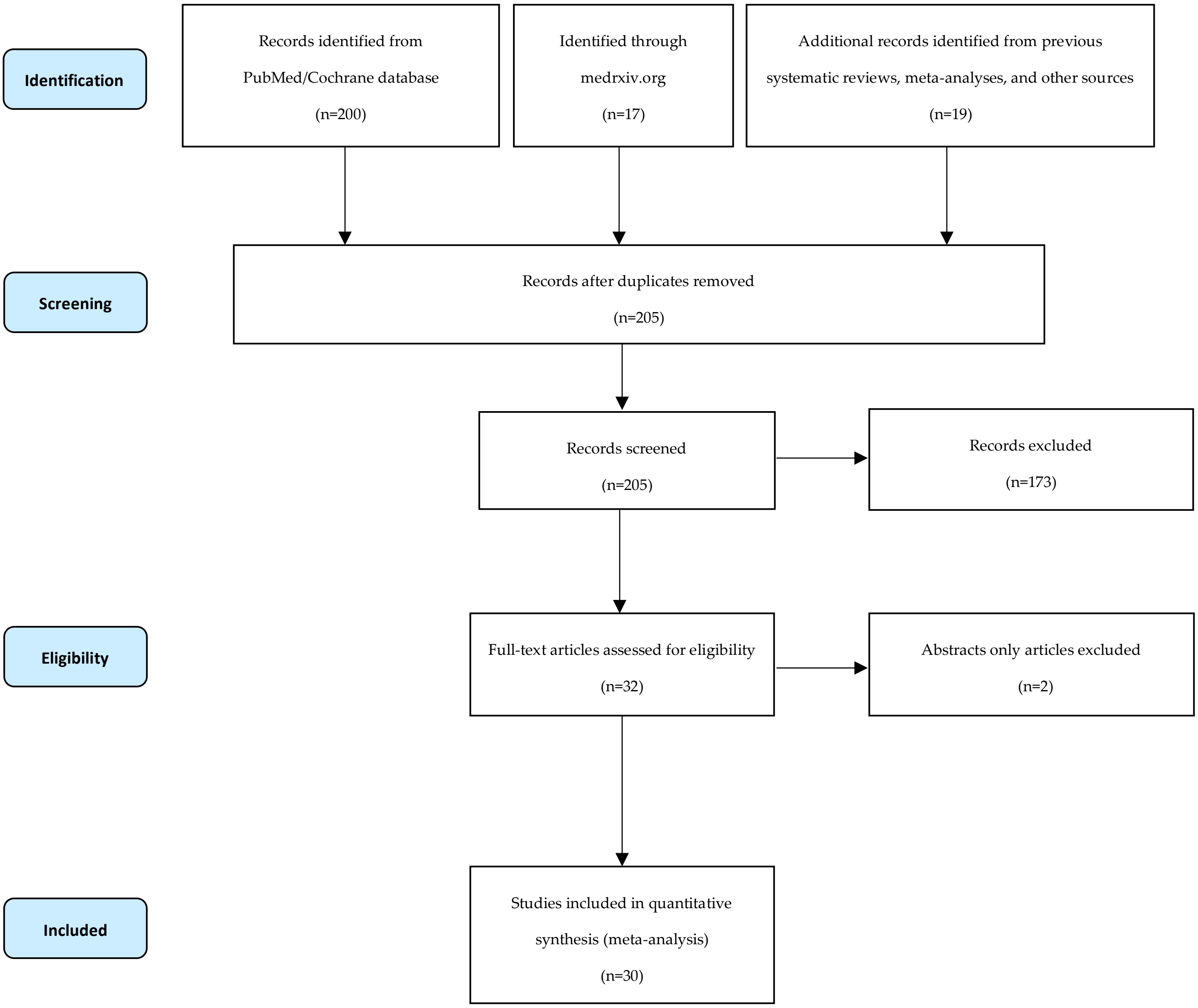

Figure Legend

Figure. S2: Funnel plots (A) mortality, (B) severity of COVID-19 disease, and (C) hospitalization.

Panel A: Mortality

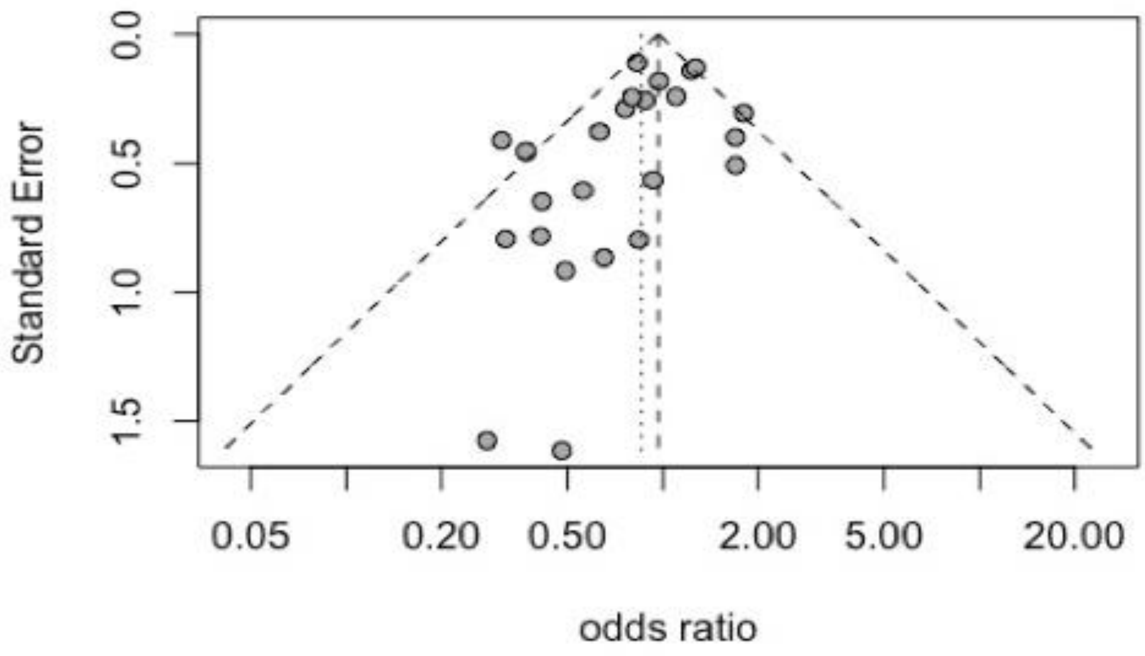

Panel B: Severity

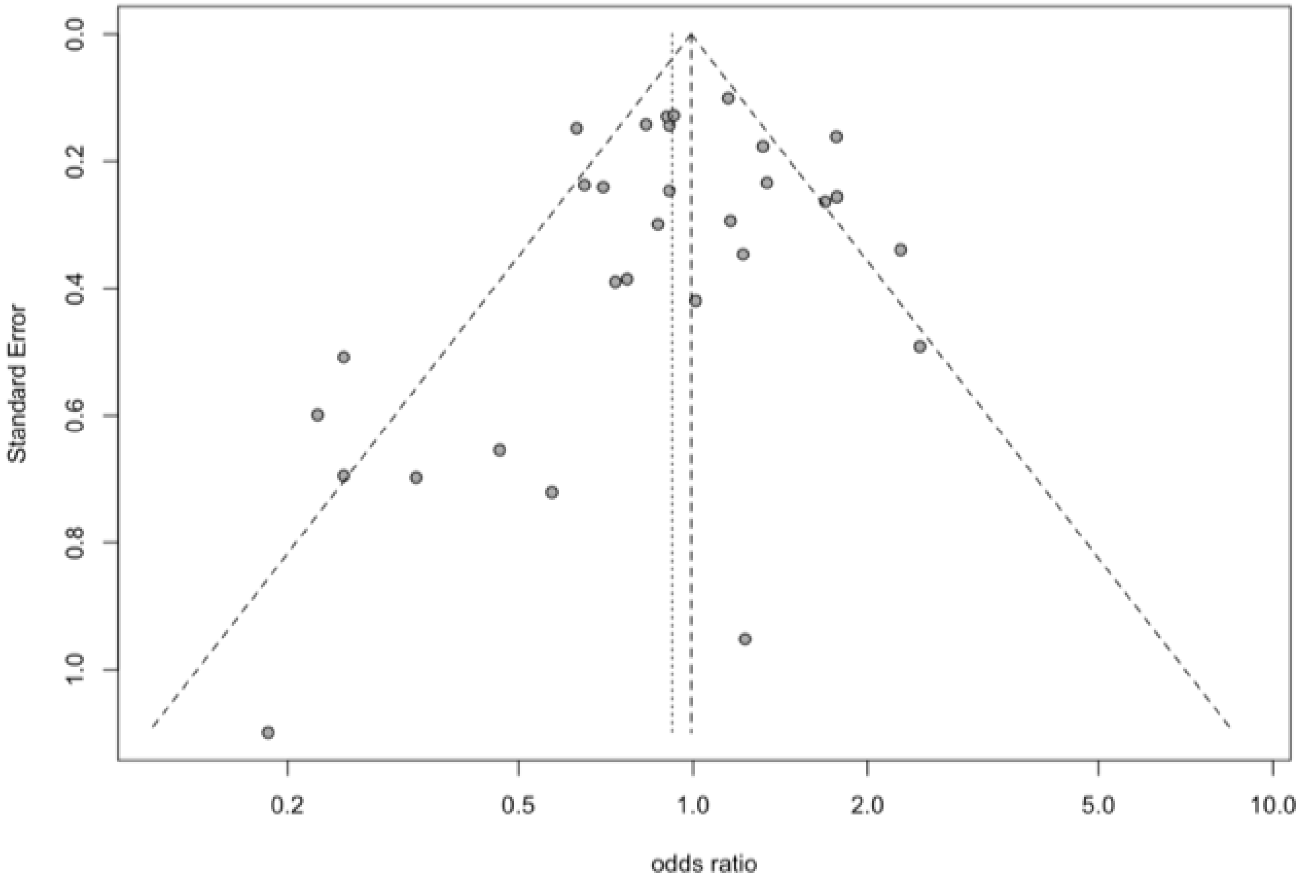

Panel C: Hospitalization

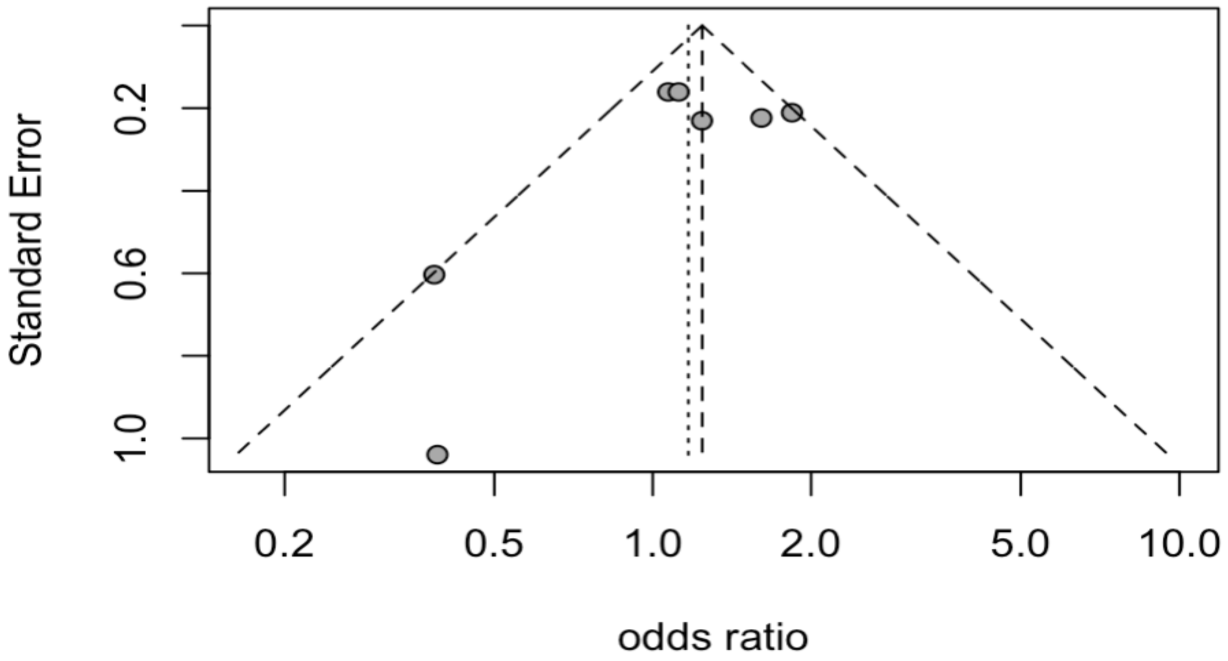

Supplement: Supplementary file 1 [file healthcare-09-00127-s001.pdf]
